# *Pseudomonas* Exotoxin A: optimized by evolution for effective killing

**DOI:** 10.3389/fmicb.2015.00963

**Published:** 2015-09-15

**Authors:** Marta Michalska, Philipp Wolf

**Affiliations:** Department of Urology, Medical Center, University of FreiburgFreiburg, Germany

**Keywords:** *Pseudomonas aeruginosa*, *Pseudomonas* Exotoxin A, virulence factor, ADP ribosylation, cytotoxic pathways, pathoadaptation

## Abstract

*Pseudomonas* Exotoxin A (PE) is the most toxic virulence factor of the pathogenic bacterium *Pseudomonas aeruginosa*. This review describes current knowledge about the intoxication pathways of PE. Moreover, PE represents a remarkable example for pathoadaptive evolution, how bacterial molecules have been structurally and functionally optimized under evolutionary pressure to effectively impair and kill their host cells.

## Introduction

*Pseudomonas aeruginosa* is a common Gram-negative, rod-shaped bacterium, which is optimally adapted in various environmental conditions. As an obligate respirer, it can use aerobic respiration as its optimal metabolism; however, it can also respire anaerobically on nitrate or other alternative electron acceptors (Su and Hassett, 2012). This is one reason, why *P. aeruginosa* is ubiquitously present in soil, water or sewage as well as in human, animal or plant hosts and why it is widespread around the world (Wiehlmann et al., 2007; Pirnay et al., 2009). Infection of healthy individuals by *P. aeruginosa* is very rare, but as an opportunistic bacterium it often colonizes immunocompromised patients with cystic fibrosis, burns, or AIDS (Gellatly and Hancock, 2013). The infections range from endophtalmitis, endocarditis, meningitis, and septicemia to chronic lung infections (Driscoll et al., 2007; Gomez and Prince, 2007; Gellatly and Hancock, 2013). Due to its inherent resistance to different antibiotics or chemotherapeutic agents, *P. aeruginosa* can only be eliminated with difficulty and leads to a high mortality rate (Maschmeyer and Braveny, 2000; Rowe et al., 2005).

**FIGURE 1:**
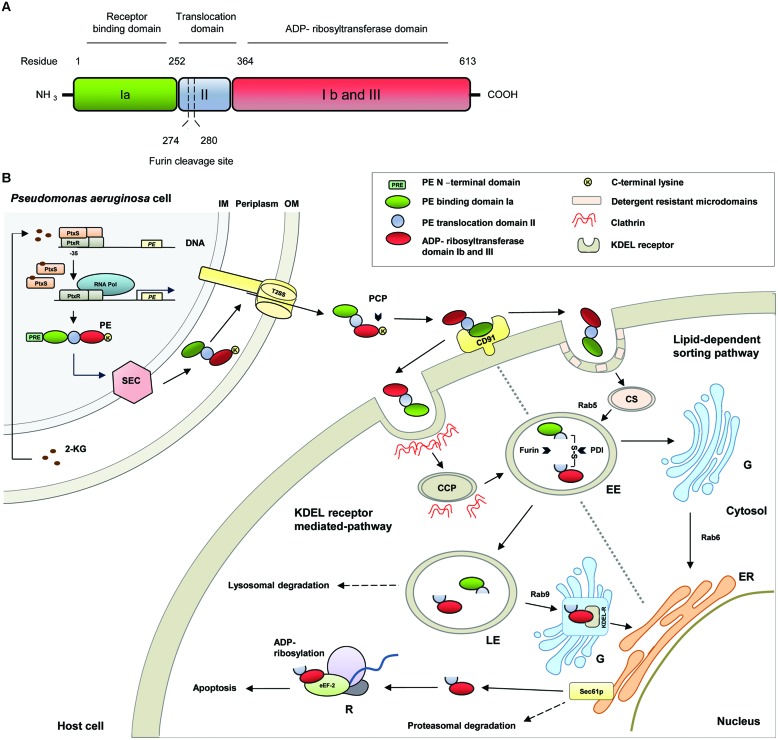
**(A)** Schematic representation of the structural and functional domains of *Pseudomonas* Exotoxin A (PE). **(B)** Molecular pathways of PE. 2-KG, 2-ketogluconate; CCP, clathrin coated pit; CD91, CD91 receptor; CS, caveosome; EE, early endosome; eEF-2, eukaryotic elongation factor-2; ER, endoplasmatic reticulum; G, Golgi apparatus; KDEL-R, KDEL-receptor; PCP, plasma carboxypeptidases; PDI, protein disulfide isomerase; PtxR, PtxS, transcription regulators; R, ribosome; Rab, Rab-GTPase; RNA Pol, RNA polymerase; Sec61p, Sec61p translocon; T2SS, type II secretion system.

A number of virulence factors enables *P. aeruginosa* to adhere to tissue surfaces, to damage tissue for dissemination and nutrition supply and to increase its survival rate (Coggan and Wolfgang, 2012; Jimenez et al., 2012; Balasubramanian et al., 2013). One of them is *Pseudomonas* Exotoxin A (PE), which has enzymatic activity and belongs to the mono-ADP-ribosyltransferase family (Liu, 1974). With regard to its function it is specified as NAD^+^-diphthamide-ADP-ribosyltransferase (EC 2.4.2.36) (Domenighini and Rappuoli, 1996). In the last years, the cytotoxic pathways of PE in eukaryotic host cells were investigated. Much relevant knowledge was obtained from studies with immunotoxins, in which the enzymatic active part of the toxin, coupled to antibodies, antibody fragments or ligands, was used for targeted therapeutic approaches against different cancers. Preclinical and clinical trials with PE-based immunotoxins were reviewed elsewhere (Wolf and Elsasser-Beile, 2009; Weidle et al., 2014). In the present article, we describe the cytotoxic pathways of PE (**Figure 1**) and how this molecule was structurally and functionally optimized under evolutionary pressure to effectively impair and finally kill its host cells.

## *Pseudomonas* Exotoxin A

### Structure and Function

The PE gene was originally cloned from the *P. aeruginosa* strain PA 103 and analysis of the 5′ and 3′ flanking regions evidenced that the PE gene is translated from a monocystronic message (Gray et al., 1984). PE is expressed as a protein with a length of 638 amino acids (aa) and can be divided into several structural and functional domains (Wedekind et al., 2001; **Figure 1A**). Generally, PE belongs to the two-component AB toxin family, composed of an A domain with enzymatic activity and a B domain as cell binding subunit (Odumosu et al., 2010). In detail, PE contains a highly hydrophobic leader peptide of 25 aa at its N-terminus, which is removed during secretion. The leader sequence is followed by the receptor binding domain Ia (aa 1–252), which is composed of antiparallel ß-sheets. Domain II (aa 253–364) with six consecutive α-helices, enables the toxin to translocate across cell membranes. The last four residues (aa 400–404) of domain Ib (aa 365–404) together with domain III (aa 405–613) form the catalytic subunit of the toxin with ADP-ribosyltransferase activity (Siegall et al., 1989).

### Molecular Pathways of Intoxication

The regulation of PE expression is complex and not fully understood to date. Different studies established a relation between PE expression and iron metabolism. The efficient uptake of iron is one important factor for *P. aeruginosa* allowing the colonization of the host. For this, the bacterium produces siderophores, such as pyoverdine, low-molecular weight excreted molecules that specifically chelate iron ions with high affinity. Interestingly, in the presence of iron ions, pyoverdine was found to activate a signaling pathway for the up-regulation of PE expression (Hunt et al., 2002; Lamont et al., 2002; Cornelis and Dingemans, 2013).

Recent data also suggest that there is a link to the bacterial glucose metabolism (Daddaoua et al., 2012, 2014). As a facultative aerobic organism, *P. aeruginosa* prefers respiration as metabolism. It gains energy by transferring electrons from glucose, a reduced substrate, to oxygen, the final electron acceptor. The initial step of glucose metabolism takes place in the periplasm and includes the oxidation of glucose to 2-ketogluconate, which enters the cytoplasm to be further metabolized. 2-ketogluconate is able to bind to the transcriptional repressor protein PtxS. In the absence of 2-ketogluconate, two PtxS molecules are bound to a dimer of the regulator PtxR, which again binds to the – 35 region to the PE promotor and inhibits the transcription of PE. After 2-ketogluconate binding, PtxS dissociates from the PtxR/DNA complex and PtxR can recruit RNA polymerase to facilitate the transcription of the toxin (Daddaoua et al., 2012, 2014; **Figure 1B**).

*Pseudomonas* Exotoxin A is secreted into the extracellular medium via the general secretory pathway, a two-step mechanism, which is highly conserved in Gram-negative bacteria (Voulhoux et al., 2000; Gerard-Vincent et al., 2002). After cytoplasmatic expression as an unfolded precursor protein, PE is initially transported to the periplasm using the Sec machinery (Douzi et al., 2012). During translocation through the inner membrane, the N-terminal signal peptide is cleaved off and PE is released into the periplasmatic space. In the hydrophilic environment of the periplasm, PE is folded to a mature conformational protein in a manner that can be recognized by the type II secretion system (T2SS), specifically called Xcp in *P. aeruginosa*, for secretion into the extracellular space (Voulhoux et al., 2000; Gerard-Vincent et al., 2002). Mutagenesis experiments gave evidence that two N-terminal glutamic acid residues at the +2 and +3 positions of domain Ia as well as domain II of PE are important for folding and extracellular secretion (Lu et al., 1993). It is therefore speculated that the corresponding residues are part of a still unknown conformational secretion signal of PE for recognition by T2SS or that they are important for the appropriate presentation of such a signal (Lu et al., 1993; Voulhoux et al., 2000).

Once secreted, the terminal lysine (aa 613) of PE can be cleaved from the toxin in the extracellular environment, presumably by plasma carboxypeptidases of the host. This leads to a formation of a C-terminal motif from REDLK (aa 609–613) to REDL (aa 609–612), which enables the toxin to bind to KDEL receptors at the Golgi apparatus during subsequent intracellular trafficking (Hessler and Kreitman, 1997). On the host cell surface, PE specifically binds via domain Ia to CD91, which is also known as alpha2-macroglobulin receptor/low-density lipoprotein receptor-related protein (α2MR/LRP; Kounnas et al., 1992). Then, there are two pathways open for PE to reach the Endoplasmatic Reticulum: the KDEL-receptor mediated pathway and the lipid-dependent sorting pathway.

#### KDEL-Receptor Mediated Pathway

CD91 bound PE molecules can be internalized via clathrin-coated pits. In the acidic early endosomal environment, PE dissociates from the CD91 receptor. Moreover, it undergoes a conformational change, which makes the furin-cleavable motif within domain II (aa 274–280, RHRQPRG) accessible. The protease furin cleaves PE between the residues R-279 and G-280, in two PE fragments. The first fragment (aa 1–279) of about 28 kDa in weight consists of domain I and parts of domain II. The second one (aa 280–613) of about 37 kDa contains parts of domain II, domains Ib, and domain III and holds the ADP-ribosylation activity (Ogata et al., 1992; Wedekind et al., 2001). After furin cleavage both fragments are still connected by a disulfide bond between C-265 and C-287, which encompasses the furin cleavage site. There is evidence that there is an unfolding event, possibly under the influence of chaperones, which leads to a surface exposure of the disulfide bond. The disulfide bond is then reduced, presumably by protein-disulfide-isomerases, and the 37 kDa fragment is detached (McKee and FitzGerald, 1999). After cleavage and transport into late endosomes, the 37 kDa PE fragment exploits a Rab9-regulated pathway to reach the trans Golgi network (TGN). Rab proteins are highly compartmentalized GTPases in organelle membranes. They coordinate consecutive stages of intracellular transport, such as vesicle formation and motility, or tethering of vesicles to their target membranes (Zerial and McBride, 2001). On the TGN, the C-terminal REDL motif of PE (aa 609–612) binds to the KDEL receptor and the toxin is transported to the ER in a retrograde manner (Kreitman and Pastan, 1995; Jackson et al., 1999). The KDEL-receptor cycles between the TGN and the ER via Golgi cisternae and is originally responsible for the recycling of cellular proteins bearing KDEL or KDEL-like sequences (Cancino et al., 2013).

#### Lipid-Dependent Sorting Pathway

*Pseudomonas* Exotoxin A can also use the lipid-dependent sorting pathway to reach the ER. In this pathway, CD91 bound PE associates with detergent-resistant microdomains (DRM), which facilitates the cellular uptake of the toxin-receptor complex via caveolin-mediated internalization. The receptor-toxin complex is then transported via caveosomes into early endosomes (EE) in a Rab5-dependent manner (Smith et al., 2006). After cleavage in the EE, the 37 kDa PE fragment can reach the TGN by a pathway, which was shown to be independent from Rab9. Then a Rab6-controlled lipid-dependent sorting pathway is used for trafficking to the ER (White et al., 1999; Smith et al., 2006).

#### ER-Associated Protein Degradation Pathway

*Pseudomonas* Exotoxin A uses the cellular ER-associated protein degradation pathway (ERAD) to get from the ER into the cytosol (Ogata et al., 1990; Theuer et al., 1993). Sequences inside the PE-domain II induce the translocation of the 37 kDa fragment via the Sec61p translocon, which normally serves as a channel to dislocate unfolded or misfolded proteins for subsequent proteasomal degradation (Hazes and Read, 1997; Koopmann et al., 2000).

### ADP-Ribosylation of eEF-2

In the cytosol the 37 kDa PE fragment exerts its enzymatic activity and ADP-ribosylates the eukaryotic elongation factor-2 (eEF-2) on the ribosomes (Iglewski et al., 1977). eEF-2 belongs to the GTP-binding translation elongation factor family and promotes the GTP-dependent translocation of mRNA from the ribosomal A-site to the P-site (Proud, 1994).

**FIGURE 2:**
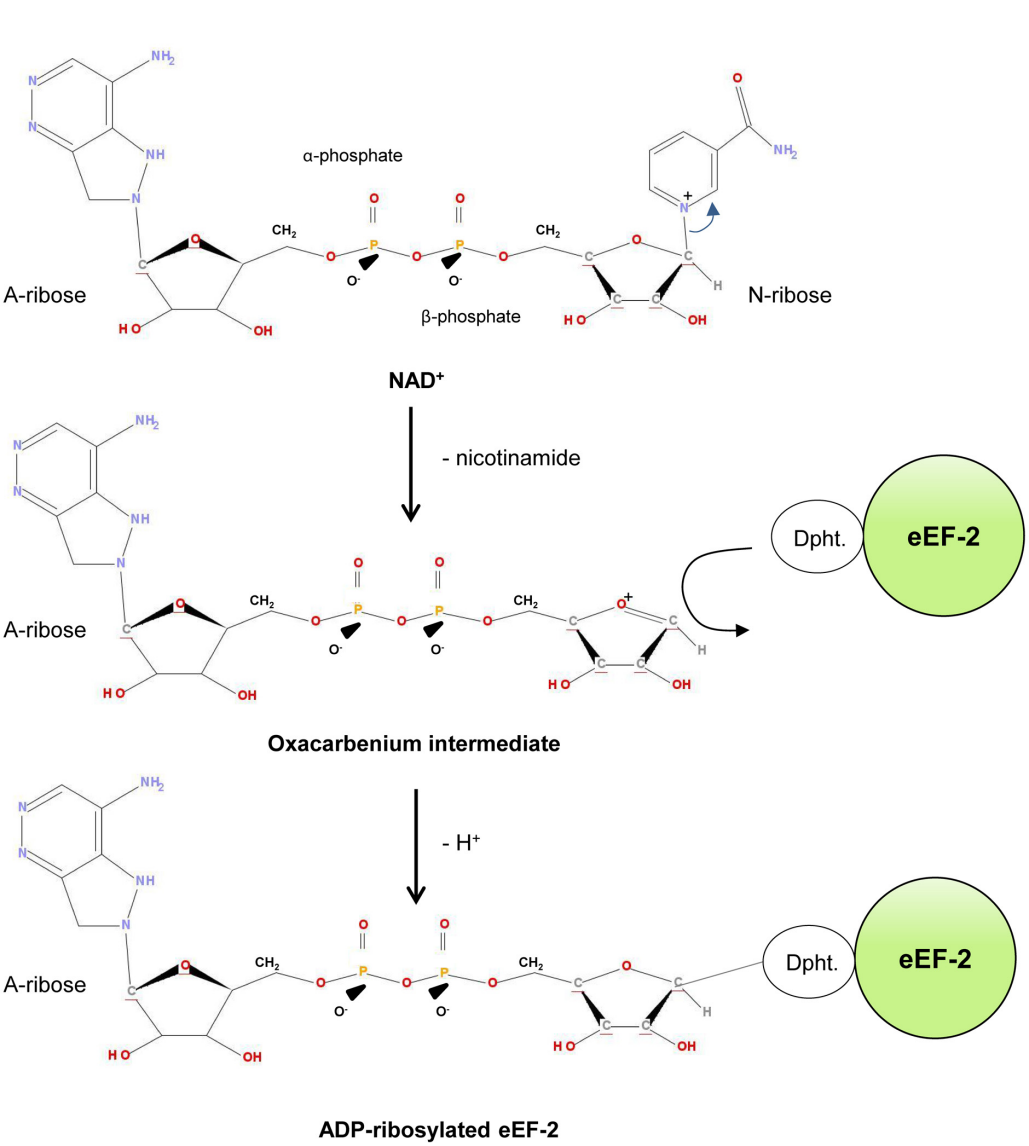
**ADP-ribosylation of eEF-2.** Dipth, diphthamide.

The ADP-ribosylation mechanism of PE was studied in detail and it turned out that it follows an S_N_1 nucleophilic substitution mechanism (Beattie et al., 1996; Armstrong et al., 2002; Jorgensen et al., 2005; **Figure 2**). Initially, the PE-fragment binds to NAD^+^ and interacts via the so-called “active-site loop L4” (aa 483–490 of domain III) with eEF-2 (Yates and Merrill, 2004). Afterward it facilicates the cleavage of the glycosidic bond (C-N) between the nicotinamide and N-ribose of NAD^+^. This results in a reactive oxacarbenium intermediate, which in turn is stabilized by residue E-553 of the PE-fragment (Li et al., 1996; Jorgensen et al., 2005). This step is followed by a nucleophilic attack of eEF-2, based on its nucleophilic residue diphthamide, a post-translationally modified histidine residue (2-(3-carboxyamido-3-[trimethylammonio]propyl) histidine) (Ortiz and Kinzy, 2005). The ADP-ribose group is subsequently transferred to the N3 atom of the diphthamide imidazole ring, which results in the ADP-ribosylated eEF-2 protein (Armstrong et al., 2002; Jorgensen et al., 2005).

The ADP-ribosylation inactivates eEF-2 and the protein biosynthesis of the host cell comes to a standstill. As a consequence, apoptosis is induced and the host cell irreversibly dies. In cervix carcinoma cells, a decrease of cdc2 and cyclin B expression as well as an increase of the regulator protein 14-3-3 delta was observed. This suggests that PE induces cell cycle arrest, which is followed by apoptosis (Chang and Kwon, 2007). In mouse embryo fibroblasts, the regulation of pro- and anti-apoptotic proteins after PE intoxication was examined. In these cells, a rapid degradation of Mcl-1 was observed, which unleashed Bak to activate apoptosis (Du et al., 2010). In human mast cells, PE provoked the activation of caspase-8 and the down-regluation of FLIPs (Fas-associated death domain protein (FADD)-like interleukin-1β-converting enzyme (FLICE) (Caspase-8) inhibitory protein), giving evidence that PE can also activate the extrinsic apoptotic pathway (Jenkins et al., 2004).

The intoxication of PE only takes a short time. In studies, which examined PE uptake into rat liver, a rapid association of PE with plasma membranes after 5–30 min, an internalization within endosomes after 15–60 min, and a translocation into the cytosolic compartment after 30–90 min was measured (El Hage et al., 2010).

The intoxication pathways of PE are not fully elucidated yet. There is evidence that there is a further processing pathway for internalized PE, involving endosomal cathepsins B and D, resulting in a production of PE fragments that may contribute to cytotoxicity (El Hage et al., 2010). Moreover, genome-wide genetic screening identified hitherto unknown host factors for intracellular trafficking. A prime example is GPR107, an orphan G-protein coupled receptor, which, like the KDEL receptor, is located to the TGN and facilitates the retrograde transport of PE (Tafesse et al., 2014). Since there are differences of PE trafficking in different cell lines, it is presumed that the choice between the pathways seems to be dependent from the expression of host factors that are present in the cells.

## Evolutionary Aspects of Intoxication

Bacteria and their hosts have coexisted for several millions of years. Over this time, bacteria developed a wide spectrum of adaptation to optimize infection and survival. One important mechanism *P. aeruginosa* developed, is the quorum sensing (QS) for intercellular communication. QS allows the bacteria to recognize the population density by sensing and measuring the accumulation of specific small signaling molecules that are secreted by the members of the colony. The bacteria now act as a community to perform tasks, which would be impossible for individual cells, e.g., cooperative activation of bacterial gene expression, biofilm formation, influence on the behavior of host cells, or the adequate production of virulence factors (Nguyen and Singh, 2006; Holm and Vikstrom, 2014).

Moreover, genome sequencing of bacterial pathogens and molecular analyses of intoxication pathways have shown how bacteria evolved via mutational changes, a mechanism, which is known as pathoadaptation.

Interestingly, the pathoadaptation of *P. aeruginosa* is exemplarily reflected in its virulence factor PE, which was structurally and functionally optimized especially in view of binding, processing, routing and toxicity (Marvig et al., 2015).

Both termini of the PE protein were formed for effective binding of target molecules. The N-terminus is able to specifically bind to the abundantly expressed CD91 antigen, which enables PE to reach many different host cells (Kounnas et al., 1992). The-C-terminus, containing the KDEL-like sequence, facilitates the retrograde transport of PE to the ER by binding to the KDEL-receptor (Kreitman and Pastan, 1995; Jackson et al., 1999).

*Pseudomonas* Exotoxin A developed specific aa motifs to be effectively processed by components of the host cell. For example, the C-terminus can be cleaved by plasma carboxypeptidases to form the KDEL-like sequence for subsequent intracellular trafficking (Hessler and Kreitman, 1997). Moreover, the molecule can be cleaved by furin, presumably to facilitate subsequent trafficking. Interestingly, the unfolding step in the EE for furin cleavage is also discussed to lead to a masquerade of the PE molecule as an unfolded/misfolded protein to be successfully transported to the cytosol (Pelham et al., 1992). PE is also able to exploit different intracellular routes controlled by both protein- and lipid-sorting signals. Especially, routing via caveasomes and the lipid-dependent pathway may contribute to protect the PE molecules against lysosomal degradation (Smith et al., 2006).

The toxicity of PE is marked by an induction of apoptosis in the host cells by specifically ADP-ribosylating the residue diphthamide. Diphthamide is named on the basis of the fact that it is also the target of Diphteria toxin produced by *Corynebacterium diphteriae* (Abdel-Fattah et al., 2013). Diphthamide is highly conserved among archaea and eukaryotes and was exclusively described in eEF-2 (Ortiz and Kinzy, 2005; Su et al., 2013). It represents an “Achilles heel” of the host cell, since its modification (ADP-ribosylation) can lead to the complete inhibition of protein biosynthesis and induction of programmed death.

Even the ADP-ribosylation mechanism of PE represents a good example for the evolutionary adaption of PE. X-ray structure analyses showed evidence that PE mimics the normal interaction between eEF-2 and the eukaryotic 80S ribosome, because a striking similarity was observed between the orientation of PE-bound ß-TAD (a non-hydrolysable NAD^+^ analog) and the phosphate backbone of two nucleotides in a conformational switch of 18S rRNA, with respect to the interaction with eEF-2. By optimizing this mimicry during evolution, PE minimizes the probability that the target organism could evolve resistance toward the invading toxin, because coordinated mutations in regions of eEF2 and the ribosome would be required that are crucial for function (Jorgensen et al., 2005).

*Pseudomonas* Exotoxin A evolved into a highly specific and toxic molecule; however, the optimization process seems to go on. Structural data suggest that there is no high-valency binding of PE to its receptor (Wedekind et al., 2001). Furthermore, lack of basic residues at the -2 aa position of the furin cleavage site could lead to less rapid cleavage by furin than proteins having the typical RX (K/R) R sequence (Gordon and Leppla, 1994). Moreover, many PE molecules are degraded in lysosomes and therefore it is necessary for the toxin to be in a sufficient concentration in the extracellular space for effective killing (Hessler and Kreitman, 1997).

Taken together, PE represents a remarkable molecule, which provides deep insight into pathoadaptive processes. Knowledge about its intoxication pathways should not only be used for the construction of PE-based immunotoxins for targeted therapy (Wolf and Elsasser-Beile, 2009; Weidle et al., 2014), but also for the development of strategies to alleviate *P. aeruginosa* infections (Jiang et al., 2014; Farajnia et al., 2015).

## Conflict of Interest Statement

The authors declare that the research was conducted in the absence of any commercial or financial relationships that could be construed as a potential conflict of interest.
